# Granulocyte Transfusion in Pediatric and Adult Neutropenic Patients: A 16-Year Retrospective Review

**DOI:** 10.7759/cureus.83578

**Published:** 2025-05-06

**Authors:** Yigit Baykara, Yaseen Jamal, AnhThu Nguyen, Thinh Quach, Suchitra Pandey, Desireny Mateo, Muharrem Yunce

**Affiliations:** 1 Pathology, Stanford University, Stanford, USA; 2 Transfusion Medicine, Stanford Health Care, Stanford, USA; 3 Technical Services, Stanford Blood Center, Stanford, USA

**Keywords:** granulocyte, granulocyte transfusion, human leukocyte antigen, neutropenia, sepsis

## Abstract

Background

Granulocyte transfusion therapy has been explored as a potential treatment for severe neutropenia, particularly in patients with life-threatening infections unresponsive to conventional therapies. However, its clinical utility remains uncertain due to inconsistent evidence, challenges in donor availability, and risks of alloimmunization. Here, we evaluated granulocyte transfusions administered to 35 pediatric and adult patients at our institution.

Materials and methods

A retrospective chart review was conducted for 35 patients who received granulocyte transfusions between 2009 and 2024. Patient data included demographics, primary diagnosis, infection type, infection site, average granulocyte count in the units, average granulocyte dose, human leukocyte antigen-calculated panel reactive antibody (HLA-cPRA) Class I and II IgG antibodies, 42- and 90-day survival, average post-transfusion absolute neutrophil count (ANC) increment, and number of transfusions. Binomial logistic regression analysis was performed to determine the clinical variables associated with increased survival.

Results

Overall survival at 42 and 90 days was 21/35 (60%) and 20/35 (57%), respectively. No significant survival differences were observed based on infection type, diagnosis, or sex. Higher body weight, increased number of transfusions, and greater granulocyte dose per kilogram were associated with improved survival. While high-dose transfusions (≥0.6×10^9^/kg) showed a trend toward better outcomes, statistical significance was not reached. HLA-cPRA Class I and II IgG antibodies correlated with lower ANC increments, though sample size limited definitive conclusions.

Conclusion

Granulocyte transfusions may benefit patients with severe neutropenia, particularly with higher doses. There are limited studies in the literature investigating the impact of HLA antibodies on ANC increment. In this study, we aimed to address this knowledge gap by providing our 16-year data from a single institution. Further prospective studies are needed to refine dosing strategies, assess the role of human leukocyte antigen alloimmunization, and optimize patient selection for improved clinical outcomes.

## Introduction

Granulocyte transfusion therapy has emerged as a potential treatment modality for patients with severe neutropenia caused by a variety of conditions, such as hematologic malignancies, chemotherapies, autoimmune diseases [1], and functional neutrophil disorders, especially those with life-threatening infections unresponsive to traditional treatment, such as antibiotics or antifungals [2]. Granulocyte transfusion, which involves the administration of donor granulocytes, aims to temporarily restore immune function and improve clinical outcomes in patients suffering from the aforementioned clinical conditions. Although strong evidence-based guidelines are lacking, in select cases, granulocyte therapy may be the best available option where the risks of untreatable infections outweigh the limitations of granulocyte therapy.

Granulocyte transfusion has been a controversial topic due to the absence of convincing data and lack of recent trials [3,4]. Early studies suggested promising outcomes, particularly in pediatric patients [5,6]. However, the most recent review that was published in 2015 [2] concluded that there were low-quality evidence to support the role of granulocyte transfusions in infection prevention and insufficient evidence to determine their efficacy in treating infections in patients with neutropenia or neutrophil dysfunction. Additionally, the most recent clinical trial, Resolving Infection in Neutropenia with Granulocytes (RING), demonstrated no survival benefit; however, post-hoc analysis suggested that patients who received high-dose granulocytes (an average dose per transfusion of ≥0.6×10^9^/kg) had better outcomes compared to the ones who received low dose [7]. However, no certain threshold is established as to the effective dose, and there was still no statistically significant difference compared to the control group.

Moreover, numerous challenges such as variability in granulocyte donor stimulation, collection methods, granulocyte dose, processing times, administration, heterogenous patient population, short shelf life, risks of human leukocyte antigen (HLA) and red blood cell antigen alloimmunization, and limited donor availability have limited widespread implementation [8,9]. Additionally, the advent of growth factors like granulocyte colony-stimulating factor (G-CSF) has provided alternative strategies to enhance neutrophil recovery, further complicating the clinical decision-making process.

Although there are several studies in the literature regarding the use of granulocyte transfusion, few have comprehensively profiled single institutional practices, and few have specifically investigated the contribution of unique factors such as neutrophil increment, HLA antibodies, diagnosis, infection type, and other procedural variables on survival. Here, we provide a comprehensive analysis of our institutional experience with granulocyte transfusion over a study period of 16 years. Broadly, we examine the impact of a wide array of clinical factors on 42-day and 90-day survival in 35 adult and pediatric patients with severe neutropenia.

The objective of this study was to evaluate clinical outcomes, including survival and absolute neutrophil count (ANC) response, in pediatric and adult patients receiving granulocyte transfusions over a 16-year period and to assess the impact of transfusion dosing and HLA alloimmunization on these outcomes.

## Materials and methods

Study design

A 16-year retrospective chart review (2009-2024) was conducted for all patients (n=35) within Stanford Medicine in Stanford, California, who received granulocyte transfusions. All adult and pediatric patients who received granulocyte transfusions, regardless of age, were included in this study. There were no exclusion criteria. Granulocyte products were collected at Stanford Blood Center (Palo Alto, California, United States) on the COBE Spectra or Spectra Optia apheresis system (Terumo BCT, Lakewood, Colorado, United States). Granulocyte donors were stimulated with dexamethasone (8 mg) 12 hours prior to collection, and hydroxyethyl starch was utilized during the procedure to increase collection efficiency. Granulocyte yield was measured by the blood center on all units using a Sysmex hematology analyzer (Kobe, Japan). All patients were premedicated with acetaminophen and diphenhydramine prior to transfusion. The following data were obtained from each patient's chart: demographics, primary diagnosis, infection type, infection site, average granulocyte count in the units, average granulocyte dose, human leukocyte antigen-calculated panel reactive antibody (HLA-cPRA) Class I and II IgG antibodies, 42- and 90-day survival, average post-transfusion ANC increment, and number of transfusions. Granulocyte products were ordered by the clinical team in communication with the transfusion services and the blood donation center. The study was approved by the Stanford University Research Compliance Office (approval number: 78820).

Statistical analysis

All statistical analyses were performed in Excel (Microsoft 365 MSO; Version 2402; Microsoft Corporation, Redmond, Washington, United States) and MATLAB (Statistics Toolbox Release 2012b, The MathWorks, Inc., Natick, Massachusetts, United States). The chi-squared test was used to determine whether the categorical variables are independent or not. The t-test was used to measure the difference between two means. Binominal logistic regression was used to determine which clinical variables are associated with increased survival. Finally, we used Pearson's correlation to demonstrate the correlation between two variables. Statistical significance was determined by a p-value below 0.05. 

## Results

Patient demographics

A total of 35 adult (57%; n=20) and pediatric (43%; n=15) patients received at least one granulocyte infusion during the study period, with 222 infusions performed in sum. The average patient age was 25.8 with a range from 2 to 63 (Table 1). The average patient weight was 59.75 kg with a range from 10.9 to 122.3 kg. Twenty patients (57%) were male (average age 31; average weight 66.7 kg), and 15 patients (43%) were female (average age, 18.9; average weight 50.4 kg). Patients' races included Hispanic (31.4%; n=11), Asian/Pacific Islander (31.4%; n=11), Caucasian (22.8%; n=8), Black (5.7%; n=2), or unknown (8.5%; n=3).

**Table 1:** Demographic characteristics of the patients

Clinical parameters	Patients (n=35)
Mean age	25.8
Mean weight (kg)	59.75 kg
Gender	
Male	20 (57%)
Female	15 (43%)
Race	
Hispanic	11 (31.4%)
Asian/Pacific Islander	11 (31.4%)
Caucasian	8 (22.8%)
Black	2 (5.7%)
Unknown	3 (8.5%)
Diagnoses	
Acute myeloid leukemia	10 (28.5%)
B-lymphoblastic leukemia	10 (28.5%)
Aplastic anemia	5 (14.2%)
Chronic myeloid leukemia	2 (5.7%)
Fanconi anemia+myelodysplastic syndrome	2 (5.7%)
Other	6 (17%)
Infection type	
Bacterial	9 (26%)
Fungal	14 (40%)
Both	12 (34%)
Site of infection	
Bloodstream	25 (71%)
Skin/soft tissue	12 (34%)
Respiratory tract	19 (54%)
Eyes	3 (9%)
Gastrointestinal tract	6 (17%)
Liver	2 (6%)
Bone	2 (6%)
Lymph node	1 (3%)

Infection, diagnosis, and survival outcomes

**Figure 1:**
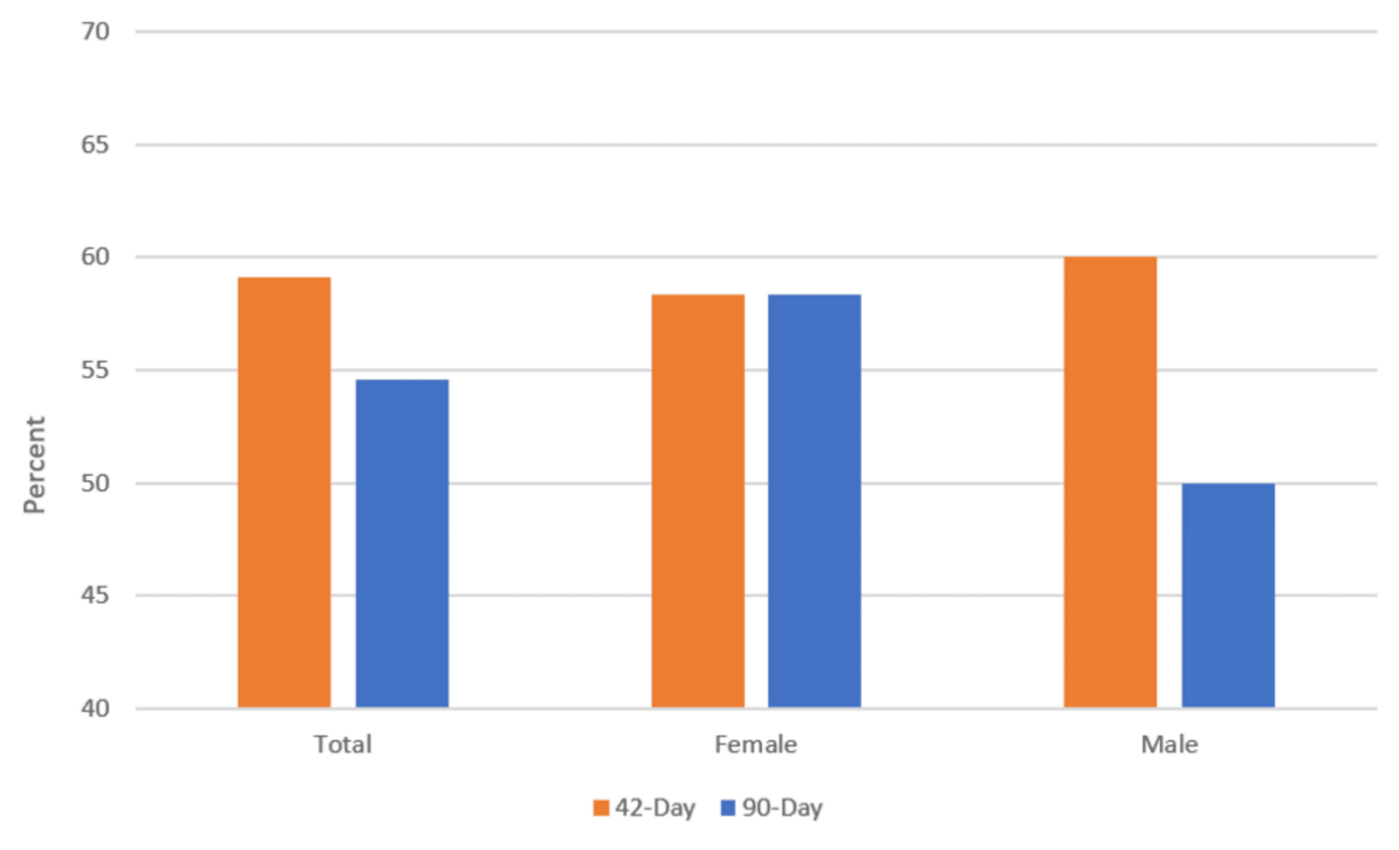
42- and 90-day (overall) survival in female and male patients

Survival was determined at 42 days [7] and at 90 days (i.e., "overall survival"). Broadly, 42-day survival was 21/35 (60%), and 90-day survival was 20/35 (57%). No difference in overall survival was observed between male and female patients (chi-squared test; p=0.77) (Figure 1).

**Figure 2:**
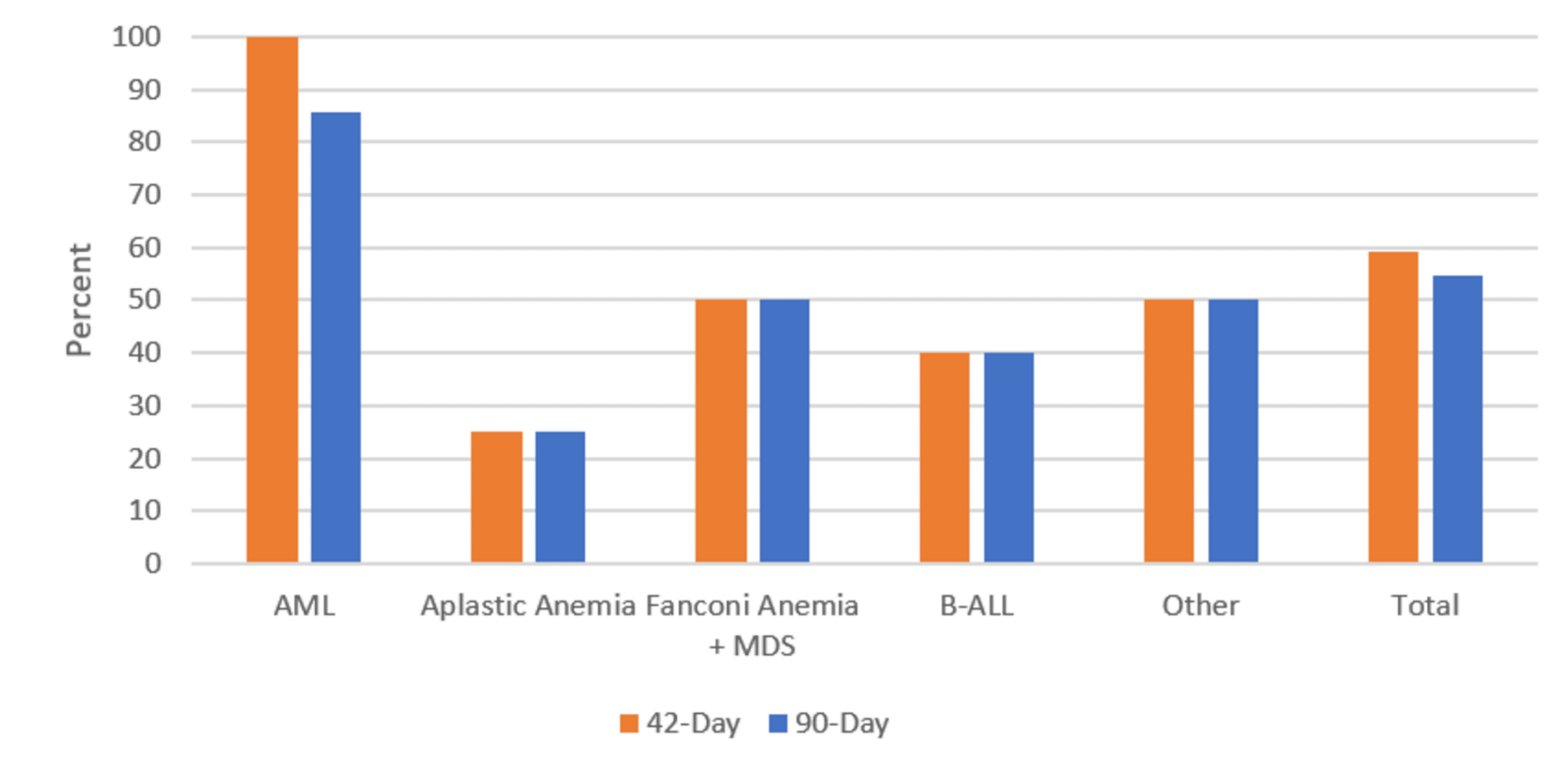
42- and 90-day (overall) survival among different diagnoses AML: acute myeloid leukemia; MDS: myelodysplastic syndrome; B-ALL: B-lymphoblastic leukemia

The most common primary diagnoses included acute myeloid leukemia (AML) (28.5%; n=10), B-lymphoblastic leukemia (B-ALL) (28.5%; n=10), aplastic anemia (14.2%; n=5), chronic myeloid leukemia (CML) (5.7%; n=2), and Fanconi anemia with myelodysplastic syndrome (MDS) (5.7%; n=2). Other diagnoses comprised six patients (17%) and included beta-thalassemia major, cystinosis, diffuse large B-cell lymphoma, diabetes mellitus type 2, NK-cell leukemia, and T-lymphoblastic leukemia (T-ALL). The highest overall survival rates occurred in patients with CML (100%; n=2) and AML (70%; n=7), with no significant difference in survival between diagnoses (chi-squared test; p=0.29) (Figure 2).

All 35 patients were diagnosed with an infection at the time of the infusion. Bacterial infections were present in nine patients (26%), and fungal infections were present in 14 patients (40%), while 12 patients (34%) had both types of infection. No difference in overall survival was observed between infection types (chi-squared test; p=0.41). The site of infection varied between patients, often involving more than one site, and 25 patients (71%) presented with bloodstream infection.

Dose, ANC response, and HLA immunization

**Figure 3:**
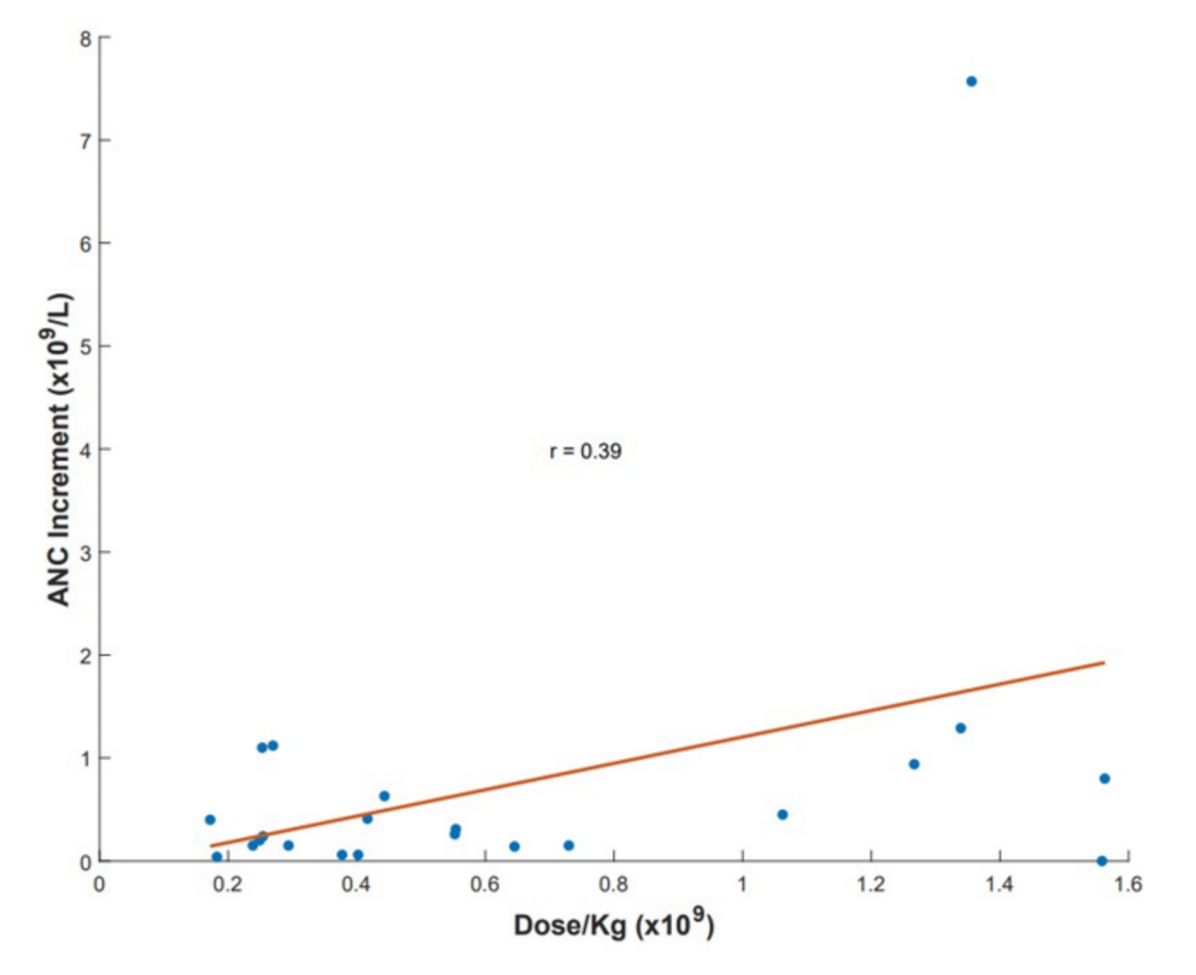
Scatter plot of post-transfusion ANC increment vs. dose by weight (R=0.39) ANC: absolute neutrophil count

On average, patients received six ABO-compatible granulocyte treatments (range 1-40). The mean dose was 21.7×10^9^ granulocytes per unit (range 12.34-64×10^9^), the mean dose by weight was 0.54×10^9^ (0.17-1.56×10^9^) per kilogram per transfusion, and the mean ANC increment post-transfusion was 0.75 K/uL (range 0-7.57 K/uL). However, 13 out of 35 patients lacked mean ANC increment data as the differential is not reported by the automated cell counter when the white blood cell count is <0.6 K/uL and manual count is not performed on every case. In addition, because of the retrospective nature of our study, there was a significant variation in the timing of post-transfusion ANC testing. High-dose treatments were defined as those receiving ≥0.6×10^9^ granulocytes per kilogram per transfusion, as previously reported [7]. Eighteen patients received high-dose transfusions; while overall survival was higher in the high-dose group, no statistically significant difference was found in comparison to the low-dose group (chi-squared test; p=0.63). Our data showed a mean post-transfusion ANC increment of 0.75 K/uL for an average dose by weight of 0.64×10^9^ per kilogram per transfusion received, excluding the dose by weight data of the patients without mean post-transfusion ANC increment data. Although technically there was a positive correlation between the dose by weight and post-transfusion ANC increment, the relationship between these two variables was weak (Pearson's correlation; R=0.39) (Figure 3).

To determine which clinical variables, if any, were significantly associated with increased survival, we performed a binomial logistic regression. Model covariates were age, weight, sex, race, diagnosis, infection type, number of transfusions, mean granulocyte dose, and mean dose by weight. HLA-cPRA Class I and II IgG levels and post-transfusion ANC increment were not included as covariates due to the limited availability of data (less than half of the patients). One-tailed significance thresholds were used for directional pre-test predictions (i.e., increased effect on the survival of treatment number, mean granulocyte dose, and mean dose by weight). Results showed a significant effect of increased weight (p<0.05; two-tailed), increased treatment number (p<0.05; one-tailed), and increased mean dose by weight (p<0.05; one-tailed) on the increase in overall survival.

**Figure 4:**
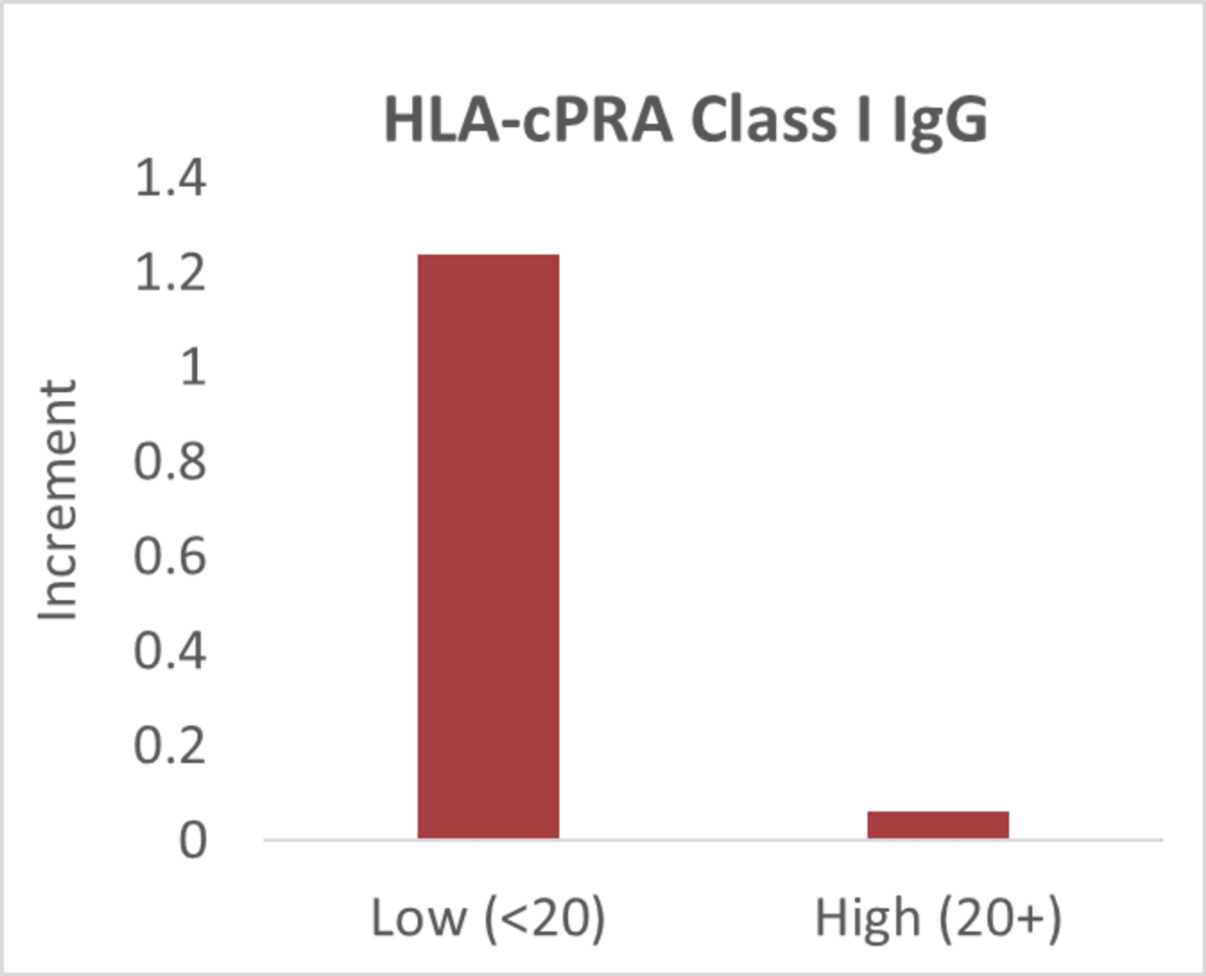
Decrease in the average increment with a high HLA-cPRA Class I IgG HLA-cPRA: human leukocyte antigen-calculated panel reactive antibody

**Figure 5:**
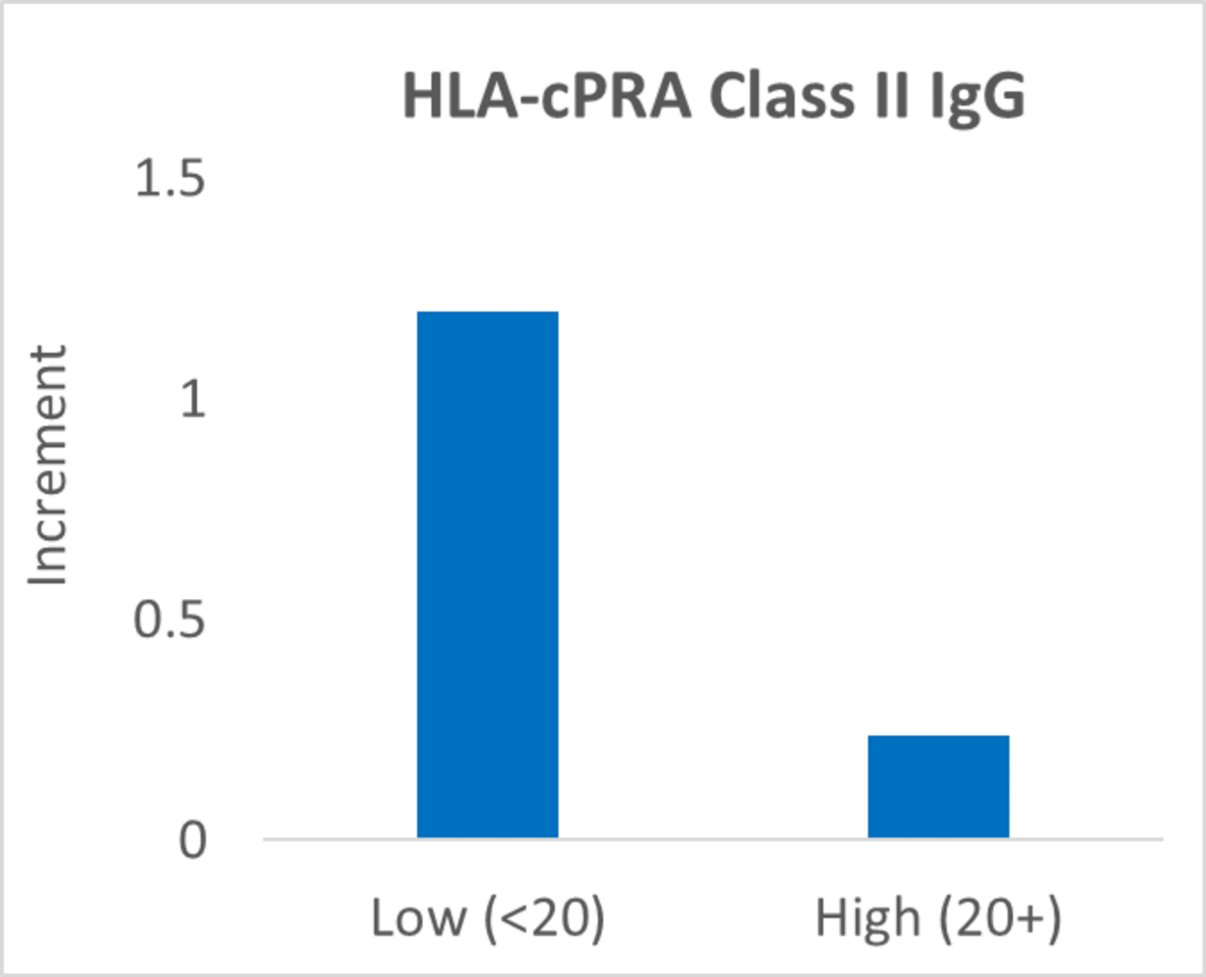
Decrease in the average increment with a high HLA-cPRA Class II IgG HLA-cPRA: human leukocyte antigen-calculated panel reactive antibody

Finally, a subset of patients (n=16) also received testing for HLA-cPRA, including Class I and Class II IgG levels. To determine the effect of HLA antibodies on ANC increment, low- and high-level groups were defined using a threshold of 20%. We derived this threshold from our institutional threshold to provide HLA-compatible platelets. We did not investigate the donor-specific HLAs as the testing was limited to the fact that most of our granulocyte donors are platelet donors and only have HLA Class I phenotype available. The average increment between the two groups was then directly compared. For HLA-cPRA Class I IgG, we observed a decrease in the average increment with a medium effect size (Cohen's d=0.5); however, this effect only approached statistical significance (t-test; one-tailed; p=0.08) (Figure 4). Similarly, for HLA-cPRA Class II IgG, we again observed a decrease in the average increment with a medium effect size (Cohen's d=0.5), and the effect was not statistically significant (t-test; one-tailed; p=0.12) (Figure 5). In view of the effect size and lack of statistical significance of these findings, we then performed a power analysis to determine the sample size needed to detect a significant effect with a power of 80%. A sample size of 27 for HLA Class I and a sample size of 41 for HLA Class II would be needed. 

## Discussion

Granulocyte transfusion has been explored for decades as a treatment option for patients with severe neutropenia and functional neutrophil disorders associated with life-threatening infections. However, its clinical utility remains a topic of debate due to inconsistent reports from multiple different studies. Moreover, its use raises important immunological concerns, notably the risk of transfusion-associated graft-versus-host disease (TA-GVHD) and potential effects on any subsequent hematopoietic stem cell transplantation (HSCT). In practice, irradiation of granulocyte transfusion units (typically 25 Gray) inactivates donor lymphocytes and is the standard method to prevent TA-GVHD. After the adoption of routine irradiation for granulocyte components, reports of TA-GVHD in this setting have become exceedingly rare. Beyond the immediate transfusion period, granulocyte transfusions can have immunological consequences that may affect a later HSCT due to HLA immunization. While granulocyte transfusions given well before an HSCT (as a "bridge to transplant" over weeks or months) carry a risk of HLA sensitization that can jeopardize donor selection and graft success [10], transfusions given during the transplant engraftment period (days immediately before or after stem cell infusion) has not shown deleterious effects on engraftment [11].

Our 16-year retrospective review provides the most comprehensive profiles of single institutional practices and a thorough analysis of a unique array of clinical factors, evaluating 42- and 90-day survival as well as the effect of HLA antibodies on post-transfusion ANCs in 35 pediatric and adult patients who received granulocyte transfusions. Overall survival at 42 and 90 days was 21/35 (60%) and 20/35 (57%), respectively. These findings are consistent with prior studies reporting survival rates ranging between 40% and 80% in patients with neutropenia receiving granulocyte transfusions [12-14].

Interestingly, our analysis did not reveal a statistically significant difference in survival based on infection type, diagnosis, or sex, suggesting that these factors may not have a significant impact on the clinical response to granulocyte transfusion. Patients who received high-dose granulocyte transfusions (≥0.6×10^9^/kg) exhibited a trend toward improved survival, aligning with post-hoc findings from the RING study [7]. However, this difference did not reach statistical significance in our cohort, potentially due to the small sample size and heterogeneity of the patient population. Nevertheless, the mean granulocyte dose per kilogram appeared as a significant predictor of survival in our logistic regression model. Despite the lack of a definitive dose threshold, these findings underscore the potential benefit of higher granulocyte doses, particularly in patients with severe infections or profound neutropenia. In addition to the mean granulocyte dose per kilogram, higher weight and increased number of transfusions were also associated with increased survival based on the logistic regression which supports the potential benefit of granulocyte transfusions. However, the increased survival seen in patients who weigh more is most likely a result of a selection bias as people who weigh less are more likely to be in a worse clinical condition and do poorly.

Subset analysis of patients with available HLA-cPRA data suggested that elevated Class I and Class II IgG levels were associated with reduced ANC increments, with medium effect sizes. However, these findings were not statistically significant due to the limited availability of HLA data. While some studies suggest that the presence of HLA antibodies has no impact on the clinical outcome [15], our results are consistent with some other studies highlighting the potential role of alloimmunization in impairing granulocyte engraftment [2,10]. Power analysis revealed that larger sample sizes are needed to definitively assess the impact of HLA antibodies, underscoring the importance of providers ordering such workups for patients undergoing granulocyte transfusion therapy. Furthermore, a standard time and manual neutrophil count when the white blood cell count is <0.6 K/uL for ANC testing are needed to assess the effects of HLA antibodies and dose by weight on post-transfusion ANC more accurately. Since our study is retrospective in nature, there was a significant variation in the timing of ANC testing and availability of data.

This study is subject to several limitations, including its retrospective design, small sample size, and heterogeneity in patient diagnoses, infection types, and treatment regimens. Additionally, the lack of standardized criteria for initiating granulocyte transfusion, the variability in post-transfusion ANC testing, and the limited availability of HLA antibody and post-transfusion ANC data restrict the generalizability of our findings. Another limitation is the lack of a control group which did not receive any granulocyte transfusions. Nonetheless, our results provide valuable insights into the factors influencing survival and treatment response in this challenging patient population.

## Conclusions

Granulocyte transfusion offers a potential lifeline for select patients with severe neutropenia and functional neutrophil disorders who are refractory to standard antibiotics and/or antifungals, particularly when high doses are administered. However, its role remains uncertain in the absence of robust, prospective evidence. There are limited studies in the literature investigating the impact of HLA antibodies on ANC increment. In this study, we aimed to address this knowledge gap by providing our 16-year data from a single institution. There is a need for a more unified HLA testing in assessing patients who may need granulocyte transfusions given the lack of response in patients with high cPRA values. Future studies should focus on optimizing dosing strategies, standardized ANC measurement timing, and HLA antibody monitoring protocols, evaluating the impact of HLA alloimmunization, and identifying predictive biomarkers of response to refine the clinical utility of granulocyte transfusions. Until then, careful patient selection and a multidisciplinary approach remain essential to maximizing the benefits of this therapy.
